# Malnutrition, Health and the Role of Machine Learning in Clinical Setting

**DOI:** 10.3389/fnut.2020.00044

**Published:** 2020-04-15

**Authors:** Vaibhav Sharma, Vishakha Sharma, Ayesha Khan, David J. Wassmer, Matthew D. Schoenholtz, Raquel Hontecillas, Josep Bassaganya-Riera, Ramin Zand, Vida Abedi

**Affiliations:** ^1^Geisinger Commonwealth School of Medicine, Scranton, PA, United States; ^2^Neuroscience Institute, Geisinger Health System, Danville, PA, United States; ^3^Clinical Nutrition, Geisinger Health System, Danville, PA, United States; ^4^NIMML Institute, Blacksburg, VA, United States; ^5^Department of Molecular and Functional Genomics, Geisinger Health System, Danville, PA, United States

**Keywords:** malnutrition, nutrition assessment tools, ASPEN, artificial intelligence, machine learning

## Abstract

Nutrition plays a vital role in health and the recovery process. Deficiencies in macronutrients and micronutrients can impact the development and progression of various disorders. However, malnutrition screening tools and their utility in the clinical setting remain largely understudied. In this study, we summarize the importance of nutritional adequacy and its association with neurological, cardiovascular, and immune-related disorders. We also examine general and specific malnutrition assessment tools utilized in healthcare settings. Since the implementation of the screening process in 2016, malnutrition data from hospitalized patients in the Geisinger Health System is presented and discussed as a case study. Clinical data from five Geisinger hospitals shows that ~10% of all admitted patients are acknowledged for having some form of nutritional deficiency, from which about 60–80% of the patients are targeted for a more comprehensive assessment. Finally, we conclude that with a reflection on how technological advances, specifically machine learning-based algorithms, can be integrated into electronic health records to provide decision support system to care providers in the identification and management of patients at higher risk of malnutrition.

## Introduction

### Malnutrition and Health

Nutrition, a vital component of proper growth and development, plays an integral role in preventing disease, maintaining health, and facilitating faster recovery. Undernutrition remains a major public health concern for people of all ages. Nutritional deficiencies of essential macronutrients, including proteins, fats, and carbohydrates, in addition to micronutrients, including vitamins and minerals (as set by the US Department of Agriculture) (1), result in various physical and psychological impairments. Current studies show that malnutrition is diagnosed in ~3–5% of hospitalized patients, but estimates indicate that the rates may be as high as 30–60% (2–5). Improvements in hospital malnutrition recognition strategies as well as collaboration between nutritionists and health care providers, could lead to better identification of patients with malnutrition. The development of targeted programs could reduce disease burden, while at the same time, improve recovery following an event, such as major surgery. One such example is the Geisinger Health System's Proven Recovery program that improves patient recovery following surgical procedures. This innovative initiative, which focuses on proper nutrition, pain management, and early mobility has seen an estimated 18% decrease of opioid use in cardiac, bariatric, spine, and joint surgical care, decrease in hospital stays by up to 50%, and an average saving of $4,556 per case for colorectal surgery patients (6). Geisinger Health System has also enhanced its approach to medicine by establishing the Fresh Food Farmacy. The Fresh Food Farmacy initiative recognizes and addresses food insecurity in local communities and promotes health through providing public education on diabetes, consultations with pharmacists and dietitians, and distribution of two free meals to families for five days (7). The Fresh Food Farmacy combats malnutrition and metabolic disorders by encouraging community members to adopt healthy behaviors and educating community members about the importance of nutrition and wellness (7).

In this work, we outline the deleterious effects of deficiencies of both macronutrients and micronutrients on the regression and recovery of neurological, cardiovascular, and immunological disorders. We summarize the assessment tools for nutrition in clinical settings. We also demonstrate how Geisinger, a large and integrated healthcare system with over one million active patients, has implemented a unified pipeline targeting malnutrition to improve care and outcome. Finally, we discuss how leveraging modeling and machine learning can help identify the most vulnerable population to better tailor care while optimizing resource utilization.

### Malnutrition and Neurological Disorders

The central nervous system and peripheral nervous system require sufficient nutritional resources to function optimally as major control centers of the body. Compensatory mechanisms during disease onset and progression rely heavily on macromolecules, coenzymes, and cofactors. Therefore, balanced nutrients are essential for appropriate recovery responses and disease regression. It is estimated that nearly 20% of patients with cerebrovascular accidents suffer from malnutrition (8). Malnutrition in stroke patients is directly associated with increased length of hospitalization and decreased rehabilitation improvements (9, 10). Mechanistically, these dietary protein-deficient patients exhibit altered genomic expression, leading to decreased hippocampal fiber plasticity and poor recovery (11). Studies on protein malnutrition have demonstrated increased hippocampal expression of trkB & GAP-43 protein, indicating increased stress response (11). In addition to macronutrients, deficiencies in micronutrients, including vitamins, are implicated in a variety of disorders and delayed recovery mechanisms (12). Vitamin B12 which is essential in the conversion of homocysteine to methionine via methionine synthase for DNA and RNA synthesis (13), produces a large number of biological agents, including the myelin sheath. It has been shown that deficiencies in Vitamin B12 are associated with higher latencies in visual evoked potentials (VEP) in patients with multiple sclerosis (14). Additionally, deficiencies of Vitamin B1, which is an essential cofactor in many cellular enzymes involved in glucose metabolism, results in energy inadequacy, and presents with similar symptomatology as Alzheimer (15). Identifying patients at higher risk of malnutrition will help design care path that is most tailored to the individual needs. This encompasses both dietary considerations with medications, increasing the chances of successful recovery and reducing the likelihood of certain neurological conditions while improving the health and well-being of patients.

### Malnutrition and Cardiovascular Disorders

Adequate intake of nutrition is important for blood vessel integrity, proper cardiac function, and sufficient myocardial mass (16). Vitamin B12 deficiency can lead to a fatal accumulation of unmetabolized homocysteine substrates, resulting in detrimental peripheral vasculature effects (17). These effects include loss of peripheral endothelium integrity, continuous vasoconstriction, and chronic inflammation (17, 18). In addition to these peripheral effects, micronutrient deficiency is also implicated in abnormal heart function and heart disease (19). Vitamin D deficiency has been shown to result in arterial stiffening, left ventricular hypertrophy, and hyperlipidemia (19).

**Figure 1:**
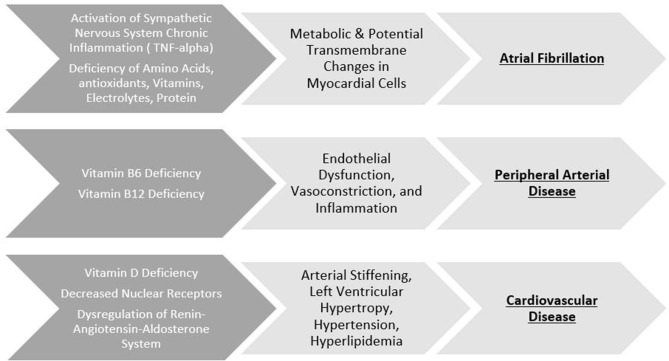
Specific nutritional deficiency and its cardiovascular outcome. **Top row**: Macronutrient and micronutrient deficiency induce various pathological changes to electrical properties leading to the development of atrial fibrillation. **Middle row**: B-vitamin deficiency disrupt the integrity of endothelial properties leading to the development of peripheral artery disease. **Bottom row**: Deficiencies of Vitamin D can result in pathological changes to the heart leading to cardiovascular disease.

Nutritional macromolecules can impact the electrophysiological properties of the heart (for example, calcium and sodium, which assist in the regulation of blood pressure and contractile properties of cardiac tissue, respectively), leading to the development of atrial fibrillation. Paradoxically, atrial fibrillation is associated with both underweight and overweight patients (20). In the case of malnutrition, the endotoxic neutralization properties of cholesterol and lipoproteins are reduced (21, 22). Additionally, loss of muscle mass is associated with increased levels of TNF-alpha, seen in atrial fibrillation (23). In the case of overnutrition, the pericardial adipose cells secrete adipokines, which alter the arrhythmogenic properties of the heart and decreases release of atrial natriuretic peptides (24, 25). (Figure 1) provides an overview of the link between specific nutritional deficiency and its cardiovascular outcome.

### Malnutrition and the Immune- Mediated Disorders

Malnutrition is associated with immune dysfunction, characterized by chronic inflammation and infections (26). Immunological responses, including activation of the immune system and secretion of inflammatory mediators, are heightened during malnutrition and can result in derangements of metabolic processes and hormone regulation (27–29). Malnutrition, in the context of inflammatory bowel disease, is bidirectional in nature. In patients with inflammatory bowel disease, nutritional deficiencies impact the cytokine profiles, which contribute to the loss of epithelial integrity and impairment in the ion transport systems (30). In turn, these changes during malnutrition and inflammation lead to exacerbation of malnutrition (30). Similarly, patients afflicted with chronic pulmonary obstructive disorder exhibit inflammatory mediators and hormones that play a role in enhanced energy utilization and decreased physical activity (31).

Patients with nutritional deficiencies have depressed immune systems and are at a higher risk for infections. Specifically, malnutrition impacts the function of hematopoietic and lymphoid organs, including bone marrow, thymus, and spleen (32). When there is a lack of protein and zinc sufficiency, the thymus can undergo atrophy and result in the death of CD4 and CD8 T cells (33, 34). Further, changes in cytokine profiles contributes to immunological alterations and immune system dysfunction. Adipose-derived from bone marrow (35) and dying spleen cells (36) secrete mediators that can alter appropriate immune responses and immune organ function. Blood collected from children with malnutrition showed decreased number of dendritic cells (37). Refer to (Table 1) for a complete list of immunological impact from malnutrition.

**Table 1:** The impact of malnutrition on immunological organs.

**Organ**	**Changes**	**Cellular**	**Signaling**	**Reference**
Thymus	Thymic atrophy	Thymocyte depletion Extracellular matrix alteration	Reduced Thymic hormone production	(38–40)
Bone marrow	Bone marrow atrophy Expansion of extracellular matrix	Megaloblastic and dysplastic changes with erythroid-series hypoplasia Loss of cellular proliferation Loss of bone marrow granulocytic cell maturation and response to CSF and LPS.	Altered cytokine microenvironment	(40–43)
Blood		Reduced Number of neutrophils, lymphocytes, and monocytes		(35)
Spleen & lymph nodes	Small spleen Thickened capsule	Reduced proliferation of spleen cells	IL-2 reduction and IL-10 production	(44)
Gut-associated lymphoid tissue	Diminished Peyer's patches and mesenteric lymph nodes	Increased NK cells	Reduced IgA in jejunal mucosa Increased TNF-alpha, IFN-y in duodenum intraepithelial lymphocyte	(45, 46)

### Malnutrition Assessment Tools and Models in Clinical Settings

Malnutrition assessment tools are utilized in clinical settings to identify specific nutritional deficiencies in patients. Assessment tools enable clinicians to gain a better understanding of the patient's condition and can promote targeted treatment strategies. American Society for Parenteral & Enteral Nutrition (ASPEN) is an example of a general approach to nutrition assessment. This toolbox considers family and medical history in addition to clinical diagnosis to determine the presence of inflammation and malnutrition. Clinical signs of fever, hypothermia, tachycardia, edema and weight gain/loss are used to recognize malnutrition. The ASPEN guidelines emphasize laboratory parameters consisting of serum albumin, C-reactive protein, white cell count, negative nitrogen balance for an objective confirmation. Further, strength tests, including handgrip dynamometer and physical performances (including timed gait, chair stands, and stair tests) are employed for assessments in the elderly (47). In addition to ASPEN, Short Nutritional Assessment Questionnaire (SNAQ) and Nutrition Risk Screening (NRS) are quick and general tools for early detection of malnutrition patients (48, 49). These tools consider biometrics, including weight, body-mass-index, food intake, and age (48, 49).

As certain patient populations require a more tailored approach to malnutrition assessments, there are different toolboxes geared toward these specific patients. For example, Mini-Nutritional Assessment Short Form (MNA-SF) examines the cognitive problems of dementia and depression in the elderly (50, 51). Patients in the intensive care unit require different nutritional assessments, which considers the severity of their illness. The Nutrition Risk in the Critically Ill (NUTRIC) and the modified Nutrition Risk in the Critically Ill (mNUTRIC) both utilize Acute Physiology, Age, Chronic Health Evaluation II (APACHEII) parameter which determines the severity of disease in patients admitted to the intensive care units (52). Both assessment tools calculate Sequential Organ Failure Assessment (SOFA) score which tracks the patient's organ function in the intensive care unit (53). In addition to these specific tools, Generated-Subjective Global Assessment (PG-SGA) is an example of an assessment tool specific to oncology patients that utilizes patient symptoms for nutrition calculation. Symptomology in cancer patients, including vertigo and nausea, negatively impact the nutritional status and improvements in these symptoms will enhance the patient's overall nutrition status (54). Refer to (Table 2) for a complete listing of general and specific nutritional assessment tools and their applications.

**Table 2:** General and specific nutritional assessment tools and their applications.

**Assessment tool**	**Data elements**	**Application**	**References**
Malnutrition Screening Tool (MST)	Weight Food Intake	Useful in acute hospital setting Useful in residential aged care setting Reliable for nutritional risk assessment of patients with pulmonary TB	(55, 56)
Mini-Nutritional Assessment-Short Form (MNA-SF)	Weight Food intake BMI Acute disease Mobility Dementia Depression	Valid nutritional screening tool for geriatric health care professionals	(50)
Nutritional Risk Screening (NRS)	Weight Food Intake BMI Disease Age	Valid tool to assess malnutrition risk in hospitalized adult population	(48)
Short Nutritional Assessment Questionnaire (SNAQ)	Weight change Appetite Supplements/tube feeding	Early detection and treatment of malnourished hospital patient	(49)
Simplified Nutritional Appetite Questionnaire (SNAQ)	Food intake Appetite Satiety Taste change	Used to evaluate the appetite loss and predict the weight loss in patients with liver cirrhosis.	(57)
Generated-Subjective Global Assessment (PG-SGA)	Weight/Weight Loss Food Intake Symptoms Activities & Function Metabolic Demand Physical Examv	Easy tool for nutrition assessment tool in hospitalized cancer patients. Allows for easy and fast identification and prioritization.	(54)
Nutrition Risk in the Critically Ill (NUTRIC)	Age APACHE II^*^ SOFA Score^¶^ Number of Comorbidities Days in Hospital IL-6	Identify critically ill patients who may receive benefit from aggressive nutritional therapy Indicator of morbidity and mortality in postoperative surgical patients	(51, 58)
Modified Nutrition Risk in the Critically Ill (mNUTRIC)	Age APACHE II SOFA Score Number of Comorbidities Days in Hospital	Good nutritional risk assessment tool for critically ill septic patients.	(51)
American Society for Parenteral and Enteral Nutrition (ASPEN)	History & clinical diagnosis Clinical & physical examination Anthropometric data Serum albumin Serum prealbumin C-reactive protein Total lymphocyte count Blood glucose Negative nitrogen balance increased resting energy expenditure Dietary data Functional outcomes	Tool to understand malnutrition syndromes in adults and practical assessment for diagnosing malnutrition syndrome	(6, 47, 59–65)

* *APACHE II, Acute Physiology, Age, Chronic Health Evaluation II, is based on 12 physiological data elements (AaDO2 or PaO2 (depending on FiO2), Temperature (rectal), Mean arterial pressure, pH arterial, Heart rate, Respiratory rate, Sodium (serum), Potassium (serum), Creatinine, Hematocrit, White blood cell count, Glasgow Coma Scale) (52). SOFA Score, Sequential Organ Failure Assessment, is based on 4 physiological data elements (Respiratory: Pa/O2/FiO2, Nervous System: Glasgow coma scale, Cardiovascular: platelet levels, Kidney: Creatine levels) (53)*.

### Geisinger's Implementation of ASPEN

ASPEN promotes nutritional care through nutrition screening, assessment, and intervention (66). Individuals who are at risk for malnutrition or are malnourished are first identified (67). Next, an assessment is performed to examine the specific malnutrition issues present so that appropriate dietary changes can be made (67). By providing nutrition support, ASPEN enables health professionals to utilize evidence-based clinical guidelines to determine malnutrition patient cases. Specifically, Geisinger implements ASPEN recommendations for nutrition support therapy in the following ways. If a patient requires nutrition support, ASPEN prefers enteral feeding over parenteral feeding (68). This is due to the fact that enteral feeding has reduced infectious morbidity (69) and fewer septic complications (70). ASPEN recommends that nutrition support therapy should be started within 24–48 h to preserve gut integrity (71). Additionally, Geisinger recognizes that malnutrition can have clinical indications, including sepsis, hyperglycemia, and obesity (72). For individuals with sepsis, low dose feedings are recommended rather than full caloric feedings (73). While ASPEN recommends that nearly 25% of nutrient intake should be protein (74), no recommendations are available for vitamins and minerals (75). ASPEN recommends that 50–60% of nutritional requirements should be glucose and 10–30% of nutritional requirements should be fat (75). Geisinger has implemented ASPEN guidelines since 2016 and has been closely following these clinical guidelines to ensure quality care for hospitalized patients with nutritional deficiencies with plans to expand to outpatient settings. Geisinger's records indicate that ~1 out of every 10 hospitalized patient has some form of malnutrition in central Pennsylvania where the participating hospitals are located. Hospitals within Geisinger document malnutrition via best practice alert (BPA). The process begins when a patient is screened for nutritional risk by a nurse and, if indicated, is then assessed by a licensed dietitian. The dietitian performs a thorough nutritional assessment and determines the degree of malnutrition, including mild, moderate, or severe, along with its clinical presentations, including fluid accumulation and loss of muscle, fat, and weight. The detailed assessment information obtained by the dietitian populates the physician note and is easily accessible to the physician. Next, the physician reviewing the patient's chart will become aware of this status, which will be addressed either during the same or future encounters depending on the patient's specific needs and overall well-being. The physician must document their encounter with the patient and document how the plan of care has been developed to treat malnutrition. Over the past year Geisinger has been working toward improving their malnutrition documentation process, by using Epic technology to incorporate SmartLinks in the SmartTexts of provider notes. These improved documentation systems have allowed Geisinger to meet the requirements set forth by Centers for Medicare and Medicaid Services, including classifying, characterizing, and planning care for patients with malnutrition. The goal is to recognize a patient who is at risk for or has malnutrition and investigate specific nutritional deficiencies and promote well-being.

**Figure 2:**
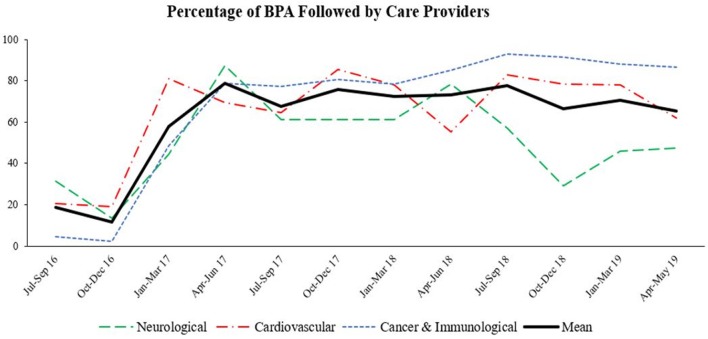
Percentage of BPA followed by care providers. Inpatient cases of malnutrition were marked as best practice alert (BPA). Percentage of BPA that was targeted for further in-depth nutritional assessment by healthcare providers in different departments among five Geisinger hospitals between July 2016–May 2019 was determined.

We have collected clinical data from the various departments across five Geisinger hospitals in central Pennsylvania. As shown in (Figure 2), the percentage of BPA that was followed by healthcare providers differed by the department. Our data shows that BPA followed through in neurology and cardiology departments fluctuated whereas BPA followed through in cancer and immunology departments remained consistent between 2017 and 2019. Furthermore, at its inception in July 2016, the percentage of physicians addressing the nutritional needs of the patients was very low (between 5 and 25%); however, once the representation of the alert in the system was refined through usability assessment, the utilization of this resource significantly improved (25–85%). These findings show that cases involving malnutrition in cancer and immunology departments are more actively and consistently addressed. Finally, the average resource utilization (acting based on the results of the assessment tool) remained stable between 60 and 80% from 2017 to 2019.

Technological advances could also aid in the fight against malnutrition. For instance, individualized recommendations about meal plans could be one of the ways to promote healthier food-consumption behaviors (76). In addition, modeling algorithms can be designed as assistive technologies for the pediatrician to help them better detect at-risk patients and (a) advise parents or guardian regarding nutrition enhancement (77), or (b) use healthcare resources including breastfeeding and vaccinations on the most vulnerable population more effectively (78).

### Modeling-Enabled Nutritional Assessment Strategies

The nutritional status of patients can be determined by performing laboratory testing, as we have outlined above. The use of these laboratory tests can enhance healthcare delivery by providing a quick and more efficient method of detecting nutritional deficiencies and prompting physicians to begin the process of in-depth assessment and intervention. However, these methodologies often fall short in identifying a larger patient population as well as following up on these patients. Artificial intelligence (AI) offers a scalable solution to these shortcomings when addressing malnutrition. Algorithms based on AI consist of various modeling strategies, including supervised learning, unsupervised learning, deep learning, and cognitive learning. While supervised learning is useful for classification and regression (79), unsupervised learning is useful for identifying patterns in data (80). Deep learning models account for parameters when making predictions from datasets whereas cognitive learning can perform pattern recognition and language processing from datasets (79). Finally, various modeling strategies, including AI, or statistical modeling approaches, such as metamodeling based sensitivity analysis (81, 82), can be designed to pull rich longitudinal clinical parameters to mine and find patterns that could lead to actionable predictions. In a review article by Verma and colleagues, authors describe the challenges in personalized nutrition and health and use of different modeling strategies in this field (83). These innovative modeling strategies provide efficient ways for healthcare systems to implement decision support algorithms to assist their care providers for a wide range of applications, including identification of at-risk patients for malnutrition.

AI could be designed to enhance the quality of care through early diagnosis, effective and personalized care plans, and risk identification and mitigation in some patients (79). Studies on integration of technology have shown that algorithms can be developed to recommend individualized meal plans for the elderly (76) as well as provide advice on nutritional enhancement and healthcare resources for children (77, 78). Further, well designed and validated algorithms can collect and mine immense amounts of longitudinal patient data from electronic health records, including body mass index, blood pressure, and body composition to predict potential clinical outcomes and recommendations for patients affected by obesity or malnutrition (84). Machine learning algorithms can be developed by integrating multi-dimensional data, including dietary intake, physical activity, blood parameters, and gut microbiota, to make personalized predictions on glycemic responses after meals (85). These machine learning methods could help promote health on a more personalized and targeted level for patients suffering from malnutrition.

## Conclusion

Nutrition impacts patients' general health, occurrence of diseases, and hospital systems at large. Although malnutrition screening tools exist, healthcare systems are not using these resources optimally and systematically. Modeling strategies, including the metamodel sensitivity analysis or machine learning-based approaches, can help identify a larger population of patients at risk for malnutrition. Our preliminary finding from one healthcare system shows the value of engaging physicians and care providers; however, more studies are warranted before malnutrition assessment tools could become truly transformative for already complex healthcare systems.

## Author Contributions

VA and RZ designed the architecture of the study. VaS, ViS, and VA performed the searches. MS, VaS, and ViS assisted with the data collection and data analysis for the case study. DW, AK, RZ, RH, and JB-R provided critical feedback and contributed to the editing of various sections of the study. VaS, ViS, and VA wrote the manuscript.

### Conflict of Interest

The authors declare that the research was conducted in the absence of any commercial or financial relationships that could be construed as a potential conflict of interest.
